# Genetic Complexity of Crohn’s Disease in Two Large Ashkenazi Jewish Families

**DOI:** 10.1053/j.gastro.2016.06.040

**Published:** 2016-10

**Authors:** Adam P. Levine, Nikolas Pontikos, Elena R. Schiff, Luke Jostins, Doug Speed, NIDDK Inflammatory Bowel Disease Genetics Consortium, Laurence B. Lovat, Jeffrey C. Barrett, Helmut Grasberger, Vincent Plagnol, Anthony W. Segal

**Affiliations:** 1 Division of Medicine, University College London (UCL), London, United Kingdom; 2 UCL Genetics Institute, University College London (UCL), London, United Kingdom; 4 Department of Surgery and Interventional Science, National Medical Laser Centre, University College London (UCL), London, United Kingdom; 3 Wellcome Trust Centre for Human Genetics, University of Oxford, Oxford, United Kingdom; 5 Medical Genomics, Wellcome Trust Sanger Institute, Wellcome Trust Genome Campus, Hinxton, Cambridge, United Kingdom; 6 Division of Gastroenterology, University of Michigan Medical School, Ann Arbor, Michigan

**Keywords:** Inflammatory Bowel Disease, Pedigree, Complex Disease, Population Isolate, AJ, Ashkenazi Jewish, AJex, Broad Institute AJ Crohn’s disease and control exome replication data set, CD, Crohn’s disease, FiDR, first-degree relative, GM-CSF, granulocyte-macrophage colony-stimulating factor, GRS, genetic risk score, GWAS, genome-wide association study, IBD, inflammatory bowel disease, IL, interleukin, LDAK, linkage disequilibrium adjusted kinships, OR, odds ratio, RAF, reference allele frequency, SNP, single-nucleotide polymorphism, UC, ulcerative colitis

## Abstract

**Background & Aims:**

Crohn’s disease (CD) is a highly heritable disease that is particularly common in the Ashkenazi Jewish population. We studied 2 large Ashkenazi Jewish families with a high prevalence of CD in an attempt to identify novel genetic risk variants.

**Methods:**

Ashkenazi Jewish patients with CD and a positive family history were recruited from the University College London Hospital. We used genome-wide, single-nucleotide polymorphism data to assess the burden of common CD-associated risk variants and for linkage analysis. Exome sequencing was performed and rare variants that were predicted to be deleterious and were observed at a high frequency in cases were prioritized. We undertook within-family association analysis after imputation and assessed candidate variants for evidence of association with CD in an independent cohort of Ashkenazi Jewish individuals. We examined the effects of a variant in *DUOX2* on hydrogen peroxide production in HEK293 cells.

**Results:**

We identified 2 families (1 with >800 members and 1 with >200 members) containing 54 and 26 cases of CD or colitis, respectively. Both families had a significant enrichment of previously described common CD-associated risk variants. No genome-wide significant linkage was observed. Exome sequencing identified candidate variants, including a missense mutation in *DUOX2* that impaired its function and a frameshift mutation in *CSF2RB* that was associated with CD in an independent cohort of Ashkenazi Jewish individuals.

**Conclusions:**

In a study of 2 large Ashkenazi Jewish with multiple cases of CD, we found the genetic basis of the disease to be complex, with a role for common and rare genetic variants. We identified a frameshift mutation in *CSF2RB* that was replicated in an independent cohort. These findings show the value of family studies and the importance of the innate immune system in the pathogenesis of CD.

See Covering the Cover synopsis on page 573; see editorial on page 593.

Crohn’s disease (CD) is a chronic, relapsing, and remitting disease of unknown etiology characterized by inflammation of the gastrointestinal tract thought to result from an aberrant immune response to commensal microorganisms in genetically susceptible individuals.^1^ CD is highly heritable (sibling recurrence risk, 13–36^2^; monozygotic twin concordance, 30% compared with 4% in dizygotic twins).^3^ Genome-wide association studies (GWAS) including more than 42,000 cases with inflammatory bowel disease (IBD) (CD and ulcerative colitis [UC]) and more than 53,000 controls have identified more than 200 disease-associated loci.^4^, ^5^ These findings have informed our understanding of the pathogenesis of CD; however, the effect sizes of the variants are small and combined they explain approximately 14% of the disease heritability.^4^

The Ashkenazi Jewish (AJ) population is a genetic isolate estimated to have arisen from 250 to 420 individuals approximately 25–32 generations ago.^6^ AJs are enriched for mutations associated with rare Mendelian^7^ and common complex diseases (eg, Parkinson’s disease^8^), and have an approximate 4-fold increased prevalence of CD.^9^ Some CD-associated loci described in non-Jewish populations also are associated in AJs and 5 novel AJ CD loci were identified by a GWAS.^10^ These, however, are unable to account for the increased prevalence of CD in AJs,^10^ suggesting unidentified, potentially rare, AJ-specific, genetic variants.

The study of large multiply affected families has the ability to identify rare, more highly penetrant variants.^11^ However, it has not been used successfully with IBD, in part because of the small sizes of the families described: of more than 1000 families recruited by a European-wide consortium, the largest included 7 cases.^12^ An AJ family with 18 cases of IBD was described, although this family was dually afflicted with basal cell nevus syndrome^13^ and genetic analysis did not identify a mutation causing the IBD.

We report the characterization of 2 large families with CD, from the ultra-Orthodox AJ community, with 54 and 26 cases of CD. Genetic analyses included examining CD-associated GWAS variants, linkage analysis, and exome sequencing with a view to identifying novel causal mutations. This study shows the genetic complexity of familial CD with a role for both common and rare variants, including a *CSF2RB* frameshift mutation that replicated in an independent cohort.

## Materials and Methods

### Ethical Considerations

Ethical and research governance approval was provided by the National Research Ethics Service London Surrey Borders Committee (10/H0806/115) and the University College London (UCL) Research Ethics Committee (6054/001). Written informed consent was provided by all participants.

### Recruitment and Phenotyping

AJ CD patients with a positive family history were recruited from the University College London Hospital and from general practices within North London. The disease status of affected individuals (cases) was established with reference to available clinical information. The absence of disease in unaffected individuals was not confirmed.

### Pedigree Drawing

Pedigrees were drawn using the Graphviz (http://www.graphviz.org/)^14^ circo function or the R package (https://www.r-project.org/) kinship2.^15^ Pedigrees have been modified to protect the anonymity of the families while maintaining the total number and distribution of individuals.

### DNA Samples

DNA was obtained from saliva collected using Oragene OG-500 DNA self-collection kits (Genotek, Ottawa, Ontario, Canada) or as described by Quinque et al.^16^ DNA was extracted by ethanol precipitation or by using QIAamp Mini spin columns (Qiagen, Hilden, Germany).

### Genome-Wide Single-Nucleotide Polymorphism Genotyping and Quality Control

Single-nucleotide polymorphisms (SNPs) were genotyped on the Illumina HumanCytoSNPv12 (Illumina, San Diego, CA) (n = 135) or the Illumina HumanCoreExome-24 (n = 282) and called using Illumina BeadStudio. Quality control was undertaken using PLINK (v1.07, http://pngu.mgh.harvard.edu/∼purcell/plink/),^17^ removing SNPs with more than 1% missingness, minor allele frequencies less than 1%, and those with Hardy–Weinberg deviation in founders at a chi-squared *P* value less than 1 × 10^-5^, leaving 288,413 and 532,426 SNPs on the HumanCytoSNPv12 and HumanExome-24 arrays, respectively, of which 113,429 were shared. The genotypes of any SNPs showing Mendelian inconsistent inheritance were set to missing in the parents and offspring in which the conflict was observed. Familial relationships were confirmed by pairwise kinship estimates.

### Ancestry Assessment

The AJ ancestry of all individuals was confirmed using principal component analysis (see the Supplementary Materials and Methods section for more detail).

### Population Level Genome-Wide Imputation

This was performed using the HumanCytoSNPv12 and HumanCoreExome-24 data separately. SNPs were phased using SHAPEIT2 (v2r790, https://mathgen.stats.ox.ac.uk/genetics_software/shapeit/shapeit.html)^18^ with duoHMM.^19^ Imputation was performed using IMPUTE2 (v2.3.2, https://mathgen.stats.ox.ac.uk/impute/impute_v2.html)^20^ with 1000 Genomes phase 3 data as reference. Imputed SNPs with information metric (INFO) greater than 0.7 were retained and genotypes with a probability greater than 0.9 were called. Variants homozygous in both parents and missing in children were populated. Data were filtered with PLINK, removing SNPs with more than 20% missingness (a relaxed threshold to minimize data losses given the stringent imputation thresholds) and individuals with more than 10% missingness.

### Genetic Risk Score and Association Analysis Using Known CD Risk Variants

Imputed genotypes for 124 GWAS CD loci were available (of 144 examined).^4^, ^10^ The 3 main CD-associated *NOD2* variants (rs2066844/p.R702W, rs2066845/p.G908R, and rs2066847/p.L1007fsinsC) were genotyped by Sanger sequencing (as per Lesage et al^21^) or using the iPLEX Gold Assay (Sequenom, San Diego, CA). For family-based association analysis, a mixed model analysis was performed using linkage disequilibrium adjusted kinships (LDAK, v5.94),^22^ which extends a standard linear regression model by including a random effect (with covariance specified by the kinship estimated from genome-wide SNP data) designed to account for correlations owing to family relatedness. Significance was assessed using a Wald test. Genetic risk scores (GRS) were calculated with Mangrove (v1.1),^23^ assuming additivity within and between loci, except for *NOD2*. Reference allele frequencies (RAFs) and odds ratios (ORs) from Jostins et al^4^ and Kenny et al^10^ were used; for the *NOD2* variants they were calculated from Zhang et al.^24^ GRS were compared with an empiric distribution in 1000 CD cases and controls simulated using RAFs and ORs. Unaffected family members were categorized into those with and without at least 1 affected first-degree relative (FiDR). Statistical comparisons were made using the Mann–Whitney–Wilcoxon test.

### Linkage Analysis

A linkage disequilibrium–pruned informative marker set was selected for linkage analysis with AJ-specific RAFs (see the Supplementary Materials and Methods section). Input files were generated using MEGA2 (v4.7.0, https://watson.hgen.pitt.edu/mega2.html).^25^ Affected-only parametric linkage analyses were performed using SwiftLink (https://github.com/ajm/swiftlink),^26^ with a 0.5 centiMorgan map and a phenocopy rate of 5% (see the Supplementary Materials and Methods section).

### Haplotype Flow Reconstruction

The reconstruction of haplotype flow was undertaken to enable the maximum number of cases sharing a founder haplotype to be examined and to permit imputation of exome sequence variants (later). A divide-and-conquer algorithm was used in which the flow of haplotypes ascertained by Merlin (v1.1.2, http://csg.sph.umich.edu//abecasis/merlin/index.html)^27^ was reassembled across split pedigrees using pairwise identical-by-descent probabilities (see the Supplementary Materials and Methods section).

### Exome Sequencing

#### Data generation

Exome sequencing was performed on DNA from all cases available (46 in Family A and 18 in Family B) and a selection of unaffected family members and AJ controls (n = 72) (see the Supplementary Materials and Methods section). Target enrichment was performed using the Agilent SureSelect Human All Exon 50 Mb kit (Agilent, Santa Clara, CA), the BGI 59 Mb Exome Enrichment kit (BGI, Hong Kong, China), or the Agilent SureSelect Exome V4 kit. Sequencing was performed on an Illumina HiSeq 2000 (Illumina), generating 75–100 bp paired-end reads, at the Wellcome Trust Sanger Institute (Hinxton, UK), BGI, or Macrogen (Seoul, South Korea).

#### Data processing

Sequence reads were aligned to Build 37 of the reference genome using Novoalign (v3.02.08) (Novocraft, Selangor, Malaysia). Duplicate reads were marked using Picard (Broad Institute, Cambridge, MA, https://broadinstitute.github.io/picard/) tools. As per GATK^28^ (v3.5, Broad Institute, Cambridge, MA, https://software.broadinstitute.org/gatk/) Best Practices,^29^, ^30^ initial genotypes were called using HaplotypeCaller and joint calling was performed using GenotypeGVCFs, with more than 4200 samples comprising UCL Exome Sequence Consortium, a local collection of exomes from a variety of cohorts. Single-nucleotide variants were filtered using variant-quality recalibration scores. Variants with genotype quality less than 10 or depth less than 5 were excluded. HumanCoreExome-24 genotypes were incorporated where available.

#### Within-family imputation

Genotypes of family members from whom genome-wide SNP data but no exome sequence data were available (5 affected and 180 unaffected in Family A and 76 unaffected in Family B) were imputed using the reconstructed haplotype flow data (see the Supplementary Materials and Methods section). This yielded data for a maximum of 254 and 114 individuals in Families A and B, respectively.

#### Variant annotation, filtering, and prioritization

Variants were annotated using Ensembl Variant Effect Predictor (VEP) (v82, http://www.ensembl.org/info/docs/tools/vep/index.html),^31^ retaining start, stop, splice, frameshift, or stop mutations, or missense mutations predicted to be deleterious by either CAROL^32^ or Condel^33^ (in any transcript), or with a CADD^34^ score greater than 20. Variants at a frequency greater than 2.5% in any 1000 Genomes^35^ or ExAC^36^ populations or greater than 5% in 1745 unrelated individuals from UCLex or 1967 non-IBD AJs (Broad Institute AJ Crohn’s disease and control exome replication data set [AJex], see later) were excluded. Finally, variants were excluded if they were missing in more than 20 cases in Family A or in more than 10 cases in Family B. Variants observed in 60% or more of the cases within each family or the 3 main subfamilies of Family A (A0, A1, and A2) were prioritized. The analysis was restricted to the autosomes.

#### Association testing and significance assessment

For each variant, within-family, kinship-adjusted association testing was performed using LDAK,^22^ with a Wald test for significance assessment. Variants were ranked by minimum *P* value within each subfamily or family.

### Replication Cohort: AJex

The RAFs of candidate variants and independent evidence of association to CD was assessed in a cohort of 1855 CD cases and 3044 controls of genetically confirmed AJ ancestry exome sequenced by an international collaborative effort coordinated by the Broad Institute as part of the Helmsley IBD Exomes Program.

### Candidate Variant Genotyping

The *DUOX2* and *CSF2RB* variants were genotyped by Sanger sequencing (see the Supplementary Materials and Methods section).

### DUOX2 Functional Experiments

The functional consequences of DUOX2 P303R were assessed in vitro using HEK293 cells co-transfected with vectors encoding wild-type or mutant DUOX2 and DUOXA2 (required heterodimerization partner) as described by Grasberger and Refetoff.^37^ DUOX2 reduced nicotinamide adenine dinucleotide phosphate oxidase activity was stimulated by ionomycin and 12-O-tetradecanoylphorbol-13-acetate as described by Rigutto et al,^38^ and hydrogen peroxide production was measured using AmplexRed horseradish peroxidase. Surface and total expression of N-terminal epitope tagged wild-type and P303R DUOX2 were determined by flow cytometry of nonpermeabilized and saponin-permeabilized cells as described by Grasberger et al.^39^ Further details are provided in the Supplementary Materials and Methods section. Repeated independent transfections were performed for each assay. Statistical analyses were performed using the Student *t* test for single comparisons and using analysis of variance with Sidak correction test for multiple comparisons.

## Results

### Description of the Families and Phenotype

**Figure 1:**
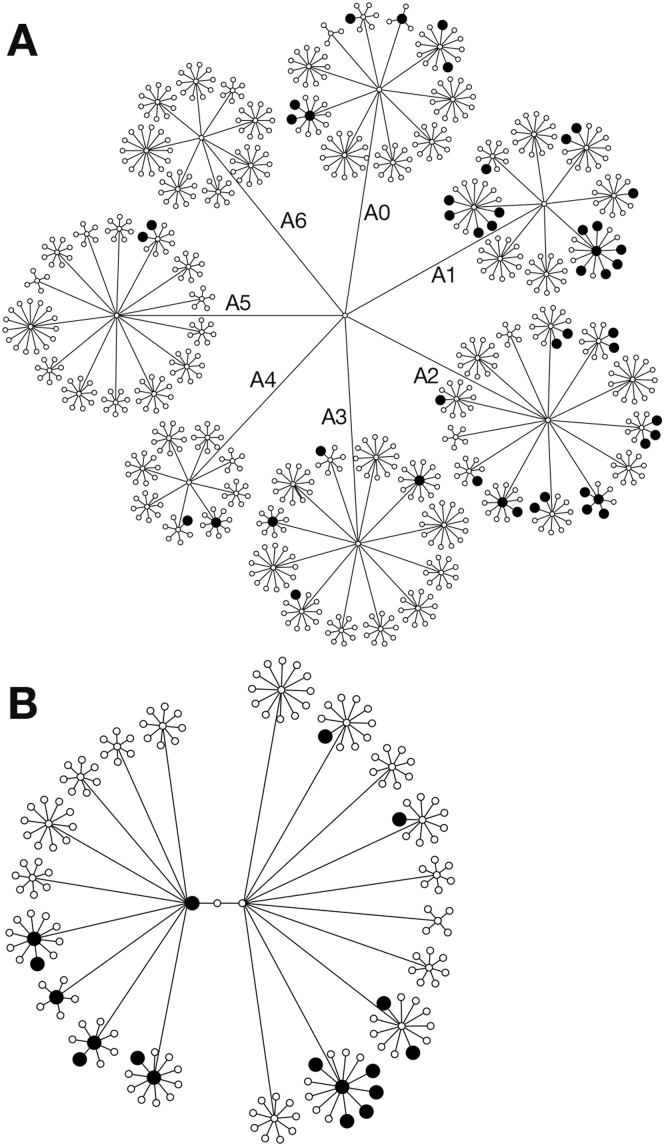
Pedigrees showing all affected (*filled symbols*, larger for clarity) and unaffected individuals in the first 4 generations of (*A*) Family A and (*B*) Family B. All individuals are shown as *circles* regardless of sex. (*A*) Subfamilies have been labeled A0–A6. The pedigrees have been modified slightly for reasons of anonymity. Deceased individuals have been included but not identified. For simplicity, founders entering the pedigrees have not been included.

**Figure 2:**
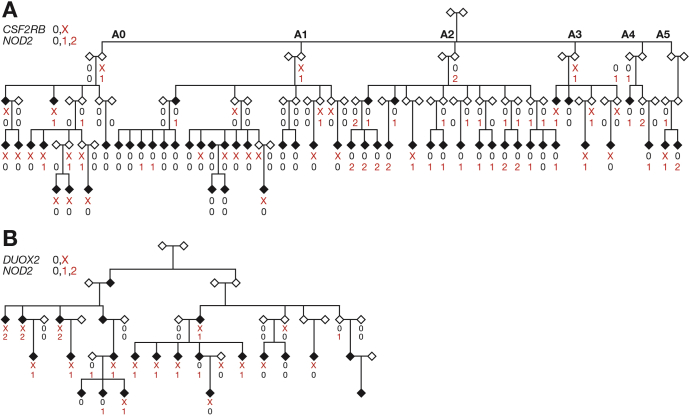
Pedigrees showing all affected individuals (*filled symbols*) and their connecting relatives in (*A*) Family A and (*B*) Family B. All individuals are been shown as *diamonds* regardless of sex. (*A*) Subfamilies have been labeled A0–A5. The genotypes of a frameshift mutation in *CSF2RB* p.S709LX22 in A, a missense variant in *DUOX2* p.P303R in B (0 wild type, X heterozygous) and a composite of the 3 *NOD2* variants examined (p.G908R, p.R702W, p.L1007fsinsC) (0, wild type; 1, heterozygous; or 2, homozygous or compound heterozygous) are shown where available.

Family A comprised a total of approximately 750 individuals across the first 4 generations including 48 cases (prevalence, 6.4%) (Figure 1*A*). There were an additional 6 cases in the fifth generation, totaling 54 (Figure 2*A*). The cases were distributed predominantly within 3 subfamilies: A0 (n = 10), A1 (n = 19), and A2 (n = 17), with prevalences of 6.9%, 15.8%, and 13.3%, respectively. The remaining cases were distributed in subfamilies A3 (n = 4), A4 (n = 2), and A5 (n = 2), with prevalences of 3.0%, 2.9%, and 1.6%, respectively (a statistically significantly lower rate, *P* < 8 × 10^-6^). Each sibship comprised an average of 10 children (quartiles, 8–11).

Individuals in Family A lived in at least 8 cities in 4 countries. There was no evidence of a bias for maternal transmission, or a sex bias (25 males, 29 females; *P* = .68). For the majority of cases (n = 48), diagnoses were confirmed by the patient’s physician or by review of their medical records. For the remaining cases, diagnoses were supported by the clinical, investigative, and treatment history. Eight cases had been labeled as UC, nonspecific colitis, or indeterminate colitis; because CD commonly is misdiagnosed as UC^40^, ^41^ and the overwhelming manifestation was that of CD, we have labeled the disease in this family as such. None of the affected individuals were cigarette smokers. The location of disease in the bowel was variable, with the majority (n = 38) having ileal or ileocolonic disease. The disease behavior included stricturing and/or fistulation in 18 cases and 14 had undergone surgical resections. The median age at onset was 18 years (quartiles, 13–21), and the minimum was 8 years. A total of 27.5% of the unaffected individuals were age 20 or younger, and a proportion of these patients are likely to develop the disease in the future.

Family B comprised approximately 180 individuals across the first 4 generations including 18 cases, with a prevalence of 9.9% (Figure 1*B*). In total, there were 23 cases within 2 subfamilies with prevalence rates of 9.8% and 10.0% (Figure 2*B*), and 3 more distantly related cases that were not included. Each sibship comprised an average of 8 children (quartiles, 6–10).

Individuals in Family B lived in at least 4 cities in 3 countries. There was a slight predominance of affected males, although this was not statistically significant (16 males, 7 females; *P* = .09). Of the 19 currently living affected individuals within the 2 main subfamilies from whom DNA was obtained, diagnoses were confirmed by the patient’s physician or by review of their medical records for all but one. One patient had severe colonic disease with nonspecific endoscopic and histopathologic features; all others had a diagnosis of CD. Five of the affected members of this family were cigarette smokers. The median age at onset was 23 years (quartiles, 16–27). A total of 8.2% of the unaffected individuals were age 20 or younger. Similar to Family A, the majority of patients had ileocolonic disease. The disease behavior included stricturing and/or fistulation in 9 cases, and 10 had undergone surgical resections.

In both families, the cases had been described as idiopathic CD (or colitis) and there were no consistently observed extraintestinal manifestations. Principal component analysis confirmed all individuals examined to be of AJ ancestry (Supplementary Figure 1). The relatedness was observed to match that expected based on the pedigree structures and there was no evidence of inbreeding. Although the pattern of disease segregation did not conform to that of a Mendelian trait, there was clear evidence of familial aggregation: in Family A, 41 of 54 cases (39 of 46 in subfamilies A0, A1, and A2) had at least 1 affected FiDR. In Family B, all but 1 of the cases had at least 1 affected FiDR.

Given the size of the families, with a population prevalence of 1.3% (a 4-fold increase of the European CD prevalence^42^) and assuming independence of disease risk using a binomial model, one would expect to observe 11 (upper 95% confidence interval, 16) and 3 (upper 95% confidence interval, 6) cases in the first 4 generations of Families A and B, respectively, compared with the 48 and 18 observed, respectively.

### The Role of Known CD-Associated Variants

Data were available for 127 CD-associated variants in 293 and 110 individuals in Families A and B, respectively. Association analysis, correcting for relatedness using LDAK, showed that 10 and 15 of these variants were nominally significant (*P* < .05) in Families A and B, respectively (Supplementary Table 1). The *NOD2* frameshift variant (rs2066847) was the most significantly associated with disease in Family A (*P* = 7 × 10^-4^), and the fifth most significant in Family B (*P* = .003).

In Family B, only 4 of 19 cases were wild type for all 3 *NOD2* variants examined (Figure 2*B*). However, in Family A, a large number of the cases (26 of 51) were wild type, predominantly within subfamilies A0 (8 of 10) and A1 (15 of 18) (Figure 2*A*). Consistent with the low penetrance of *NOD2* variants, 12 unaffected individuals in Family A and 2 in Family B were either compound heterozygous or homozygous.

**Figure 3:**
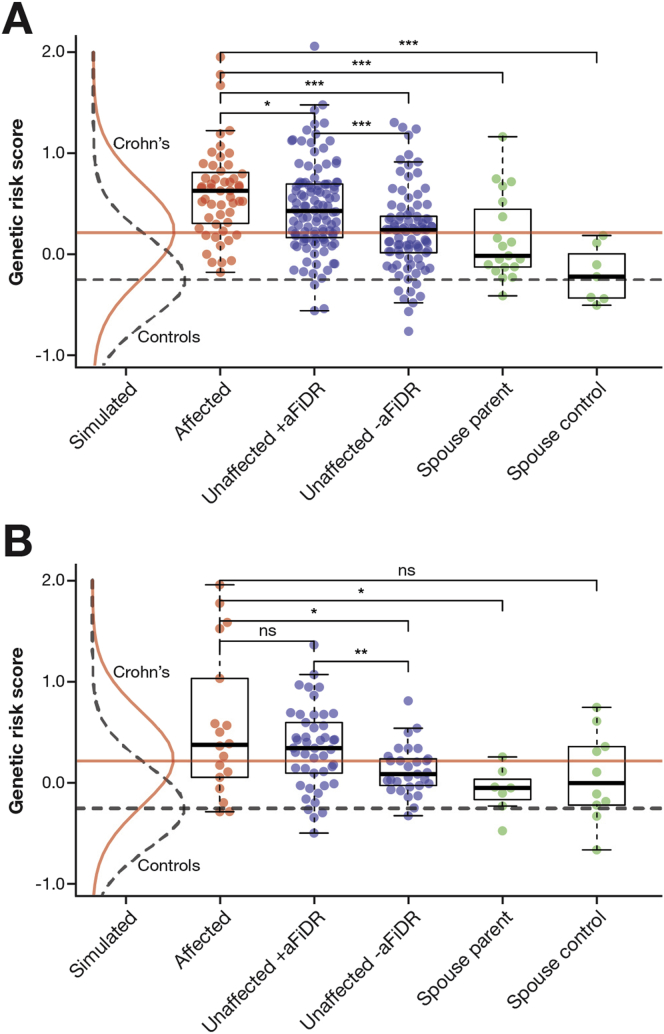
GRS in affected and unaffected individuals in (*A*) Family A and (*B*) Family B. Unaffected individuals have been divided into those with (+aFiDR) and without (-aFiDR) at least 1 affected first-degree relative. Spouse controls; founders with no affected descendants; spouse parents; founders with 1 or more affected offspring. (*A* and *B*) The distribution of GRS in a simulated theoretical control and Crohn’s disease population (along with the corresponding medians) is indicated. *P <.05, **P <.01, and ***P <.001 (Mann–Whitney–Wilcoxon test).

The cases in Family A harbored a significant burden of common CD-associated variants with an approximately 2.9-fold higher median GRS than the simulated CD population (Figure 3*A*). Cases had a higher GRS than unaffected individuals with (*P* = .038, without correction for 5 comparisons) and without (*P* = 7.4 × 10^-7^) an affected FiDR. Unaffected individuals with at least 1 affected FiDR had a significantly greater GRS than those without (*P* = 1.2 × 10^-4^). The median GRS of spouse controls (founders with no affected descendants) approximated to that of the theoretical control population. In Family B (Figure 3*B*), there was no significant difference in GRS between cases and unaffected individuals with at least 1 affected FiDR; however, unaffected individuals with an affected FiDR did have a higher GRS than those without (*P* = .009). Although the prevalence of disease was much greater in subfamilies A1 and A2 compared with A3, A4, and A5, the GRS (in both affected and unaffected individuals) was not increased proportionately (Supplementary Figure 2).

### Linkage Analysis

Parametric analyses failed to identify 1 or more loci significantly segregating with disease. The maximum logarithm of the odds (base 10) scores for Family A; Family A subfamilies A0, A1, and A2 together; and Family B were all less than 1.2. A variety of strategies were used, varying the definition of the affected status (eg, only those with histologically confirmed CD) and altering the phenocopy rate. Maximal haplotype sharing confirmed these results and showed that, at most, 26 of 50 affected individuals within Family A and 14 of 17 within Family B shared a locus identical by descent. At the *NOD2* locus, at most, 10 of 17 affected individuals were identical by descent in Family B.

### Exome Variants

A total of 68,881 exonic variants were seen in at least 1 affected individual in Family A and 52,682 exonic variants in Family B (an average of 18,221 per individual across both families). After filtering, 66 variants were prioritized in Family A and 11 in Family B (Supplementary Tables 2 and 3, respectively). No variants known to cause monogenic intestinal inflammation^43^ were observed. For both families, the minimum theoretical LDAK *P* value for a variant segregating to all affected individuals and no terminal unaffected individuals was a *P* value less than 1 × 10^-16^. Within-pedigree variant imputation was successful for 62 of 66 and 9 of 11 of the prioritized variants in Families A and B, respectively.

In Family A, 20 of the prioritized variants were enriched in affected compared with unaffected family members at a LDAK *P* value less than .05 within either the entire family or 1 of the 3 main subfamilies (Supplementary Table 2). The top 10 variants ranked by minimum *P* value across the family or in any subfamily are shown in Table 1. Similar exome findings were observed when the cases labeled as UC or indeterminate colitis were excluded. For 16 of these variants, data were available from AJex; 2 variants were nominally significantly (*P* < .05) associated with AJ CD: the *NOD2* frameshift rs2066847 (AJex *P* = 2.1 × 10^-25^) and a frameshift mutation in *CSF2RB* (p.S709LX22). The latter was observed in 18 of 40 affected and 27 of 103 unaffected individuals in the family, with a particular enrichment in subfamily A0 (8 of 9 affected and 7 of 24 unaffected; LDAK *P* = 6.1 × 10^-5^). This variant was associated with CD in AJex at a *P* value of .0091. The identical *CSF2RB* frameshift mutation was identified in a concurrently published study of 2992 unrelated CD cases and 9594 controls, all of AJ ancestry, in which it was associated with disease at a *P* value of 3.42 × 10^-6^, with an OR of 1.5.^44^ The composition of the case and control cohorts studied by Chuang et al^44^ partially overlaps with AJex; however, the discovery of this variant in Family A and by Chuang et al^44^ were independent.

**Table 1:** The Top 10 Exome Variants Sorted by Minimum *P* Value in Family A or its Constituent Subfamilies

Chromosome	Position	Ref	Alt	Gene	ExAC	A	U	P	mA	mU	mF	minP	AJexOR	AJexP
22	37333972	GC	G	*CSF2RB*	1.4 × 10^-3^	0.19	0.13	.014	0.44	0.15	A0	6.1 × 10^-5^	1.5	9.1 × 10^-3^
4	73013007	CA	C	*NPFFR2*	8.2 × 10^-5^	0.12	0.03	1.2 × 10^-4^			A	1.2 × 10^-4^	ND	ND
19	55481394	C	T	*NLRP2*	9.2 × 10^-3^	0.15	0.05	2.7 × 10^-4^			A	2.7 × 10^-4^	1.2	.26
16	50763778	G	GC	*NOD2*	0.013	0.17	0.08	1.9 × 10^-3^	0.43	0.15	A2	3.4 × 10^-4^	3.1	2.1 × 10^-25^
16	88694161	C	T	*ZC3H18*	2.0 × 10^-4^	0.22	0.13	.11	0.37	0.13	A2	1.2 × 10^-3^	1.3	.36
7	99702938	G	A	*AP4M1*	1.3 × 10^-4^	0.12	0.07	.3	0.30	0.09	A0	3.2 × 10^-3^	0.9	.91
9	136385356	C	T	*TMEM8C*	3.2 × 10^-4^	0.25	0.14	5.3 × 10^-3^			A	5.3 × 10^-3^	1.1	.83
4	69962375	T	C	*UGT2B7*	2.7 × 10^-3^	0.12	0.04	5.3 × 10^-3^			A	5.3 × 10^-3^	0.4	.23
10	120889108	A	G	*FAM45A*	4.6 × 10^-4^	0.16	0.11	.066	0.33	0.12	A0	6.3 × 10^-3^	0.8	.22
4	175225400	T	C	*CEP44*	4.0 × 10^-3^	0.30	0.21	.044	0.50	0.26	A0	7.2 × 10^-3^	ND	ND

NOTE. Variant positions are given with reference to Build 37 of the human genome. All allele frequencies reported are for the alternate allele.

A, allele frequency in cases; AJexOR, replication odds ratio; AJexP, replication *P* value; Alt, alternative allele; ExAC, population allele frequency; mA, allele frequency in cases in the subfamily yielding the minimum *P* value; mF, subfamily in which the minimum *P* value was observed; minP, minimum LDAK *P* value across all subfamilies or the entire family; mP, corresponding *P* value; mU, corresponding allele frequency in unaffected individuals; *P*, LDAK *P* value; ND, no data available; Ref, reference allele; U, allele frequency in unaffected individuals.

In Family B, 7 of the 11 prioritized variants showed enrichment in affected compared with unaffected family members at a LDAK *P* value less than .05 (Table 2). A missense variant in the *DUOX2* gene (p.P303R) was shared by the largest number of affected individuals (15 of 19) and yielded the most significant *P* value (LDAK *P* = 1.6 × 10^-4^). The second most commonly shared variant was the *NOD2* frameshift (LDAK *P* = 9.7 × 10^-3^). For all of the 7 variants achieving a *P* value less than .05 within the family, data were available from AJex; the *NOD2* frameshift and a variant in the gene *PLA2G4E* were associated at a *P* value less than .05 in these data; however, the direction of effect of the latter was opposite that seen in Family B. Considering all 3 *NOD2* variants, 12 of 19 affected individuals were heterozygous for the *DUOX2* variant and had at least 1 *NOD2* variant as compared with only 12 of 88 unaffected individuals. A missense variant in *RNF186* (rs41264113, p.A64T) recently associated with UC^45^ was observed in 12 of 19 cases, although it was not enriched in cases (*P* = .39).

**Table 2:** The Seven Prioritized Exome Variants in Family B

Chromosome	Position	Ref	Alt	Gene	ExAC	A	U	*P*	AJexOR	AJexP
15	45402883	G	C	*DUOX2*	0.011	0.37	0.15	1.6 × 10^-4^	1.2	.26
15	42281657	C	T	*PLA2G4E*	8.0 × 10^-4^	0.32	0.12	3.2 × 10^-4^	0.6	.032
5	44809369	C	T	*MRPS30*	0.011	0.29	0.15	8.2 × 10^-3^	1.1	.84
16	50763778	G	GC	*NOD2*	0.013	0.42	0.21	9.7 × 10^-3^	3.1	2.1 × 10^-25^
1	179660076	T	TGAGG	*TDRD5*	ND	0.29	0.15	.019	0.7	.25
3	48414274	C	T	*FBXW12*	0.014	0.32	0.14	.020	1.1	.50
1	117492067	T	G	*PTGFRN*	1.9 × 10^-4^	0.29	0.14	.030	1.3	.51

A, allele frequency in cases; AJexOR, replication odds ratio; AJexP, replication *P* value; Alt, alternative allele; ExAC, population allele frequency; *P*, LDAK *P* value; ND, no data available; Ref, reference allele; U, allele frequency in unaffected individuals.

There was no difference in the clinical phenotype of cases harboring the *CSF2RB* or *DUOX2* variants relative to the noncarrier affected individuals within the families.

### Functional Assessment of the *DUOX2* Variant

**Figure 4:**
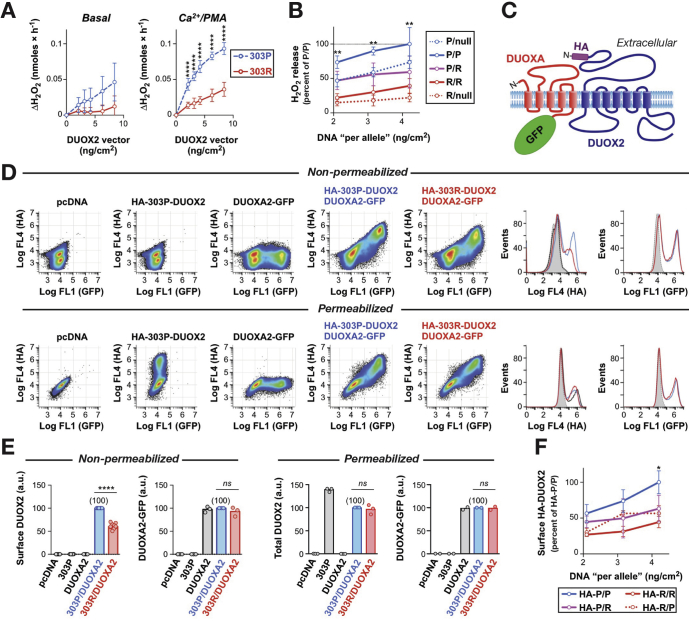
Functional characterization of the effect of DUOX2 P303R in vitro. (*A*) Hydrogen peroxide production from DUOXA2-reconstituted HEK293 cells transfected with 303P (wild type) and 303R DUOX2 at a range of vector concentrations. Data represent means ± SD of 3 (basal) and 6 (stimulated) independent experiments. The total amount of DNA per transfection was kept constant by adjusting with empty vector. ****P < .0001 (Student *t* test). (*B*) To simulate heterozygosity, cells were co-transfected with the indicated combinations of empty vector (null), DUOX2 303P, or 303R plasmids. Values represent means ± SD from 6 experiments per transfection dose. The activity of P/R (heterozygous 303P/303R) is significantly lower than P/P (homozygous 303P), but indistinguishable from P/null (monoallelic deletion 303P/null), P/R vs P/P: **P < .01 (analysis of variance with Sidak correction). (*C*) Topology model of the DUOX2/DUOXA2 complex at the plasma membrane showing the location of the introduced hemagglutinin (HA) epitope tag (DUOX2) and green fluorescent protein (GFP) fusion (DUOXA2). (*D*) Representative flow cytometry scatterplots and histograms showing the detection of the HA epitope and GFP fluorescence in cells transfected with the indicated plasmids. (*E*) Summary of DUOX2 and DUOXA2 expression assessed by flow cytometry. For each experiment (*open circles*), data are expressed relative to the value for the 303P/DUOXA2 transfection (set to 100). a.u., arbitrary units; ****P < .0001; ns, *P* > .05 (Student *t* test). (*F*) To assess the surface expression of 303P and 303R DUOX2 under heterozygous conditions, the expression of the HA epitope at the cell surface was determined in cells co-transfected with equal amounts of 2 DUOX2 plasmids, with only 1 plasmid containing an HA tag. Values represent means ± SD from 3 experiments per transfection dose. Results suggest interference of 303R with surface expression of 303P: HA-P/R vs HA-P/P: *P < .05 (analysis of variance with Sidak correction).

In DUOXA2-reconstituted HEK293 cells, hydrogen peroxide production by DUOX2 P303R was reduced significantly compared with the wild-type protein at a range of vector concentrations (Figure 4*A*). Similar findings were observed in HeLa cells (data not shown). Neither wild-type nor DUOX2 P303R resulted in significant superoxide production, indicating that lack of hydrogen peroxide production by DUOX2 P303R was not owing to deficient intramolecular dismutation of superoxide (Supplementary Figure 3). Flow cytometric analysis of N-terminal epitope-tagged DUOX2 showed that the reduced activity of P303R could be accounted for by a failure to efficiently traffic the variant protein to the cell surface despite normal intracellular expression level (Figure 4*C–E* and Supplementary Figure 4). Simulation of heterozygous conditions by co-expression of DUOX2 P303R with the wild-type protein showed partial interference of the variant protein with the surface expression and function of wild-type DUOX2 (Figure 4*B* and *F*). Based on these in vitro findings, the severity of DUOX2 loss in heterozygous carriers is predicted to be equivalent to a monoallelic deletion mutation.

## Discussion

This study identified 2 large families with CD. However, even in such large families and despite a plethora of genetic data, disentangling the causes of the disease proved challenging.

In both families there was a considerable burden of GWAS CD-associated risk variants in both the affected and unaffected individuals. In Family A there was a marginal increase in GRS in affected individuals compared with unaffected individuals, with at least 1 affected FiDR; however, no such difference was observed in Family B. Furthermore, in Family A the GRS did not correlate with prevalence by subfamily, suggesting the presence of additional etiologic factors in those subfamilies with a higher prevalence. In the absence of AJ-specific RAFs and ORs for all risk variants and an accurate AJ population disease prevalence, the role of common variation in the familial aggregation cannot be quantitated with precision; however, it is likely to contribute significantly.

Linkage analysis is hampered by the presence of phenocopies (cases with a different genetic cause for their disease), which in these families are probable given the high prevalence of CD in the AJ population and the family sizes. The absence of linkage does not, however, exclude the possibility that a large subset of the cases within each family (or subfamily) might share a rare damaging variant, as exemplified by the *NOD2* frameshift, which was prioritized successfully by exome sequencing. However, no exome variant (including the *NOD2* frameshift) met the minimum theoretical *P* value or achieved genome-wide significance (*P* < 5 × 10^-8^).^46^

The lack of within-family, genome-wide significance may be overcome by using independent replication cohorts. This, however, requires large sample sizes in the case of rare variants.^46^ Nonetheless, nominal significance was obtained for 2 variants, the *NOD2* and *CSF2RB* frameshifts, the latter at a *P* value of .009. Theoretically, it would be appropriate to impose a Bonferroni significance threshold of *P* less than .0025 (20 variants tested). However, importantly, a concurrently published study independently discovered the identical *CSF2RB* frameshift mutation in the context of AJ CD and provided strong statistical evidence for the association. Furthermore, functional investigations have shown that this variant impairs STAT5 phosphorylation after granulocyte-macrophage colony-stimulating factor (GM-CSF) stimulation both in transfected HEK293 cells and in monocyte-derived macrophages isolated from carriers.^44^ Homozygous loss-of-function mutations in *CSF2RB* have been described in pulmonary alveolar proteinosis, in which there is completely absent GM-CSF–induced STAT5 phosphorylation.^47^, ^48^ None of the patients in our study with the *CSF2RB* frameshift showed a pulmonary phenotype, presumably because of the existence of some residual CSF2RB activity.

CSF2RB is an excellent candidate for causal involvement in the pathogenesis of CD: the protein forms the β chain of the interleukin (IL)3, IL5, and GM-CSF receptors, which signal through STAT5 to influence the differentiation, proliferation, and function of hematopoietic cells. Loss of GM-CSF signaling is associated with compromised immunity.^49^ This is pertinent in the context of CD as an immunodeficiency disease in which the innate immune system fails to adequately clear microorganisms that have breached the mucosal barrier, owing to impaired cytokine secretion causing defective neutrophil recruitment, with a resulting secondary inflammatory reaction.^50^, ^51^, ^52^, ^53^

Although not significant in AJex despite sufficient power given its RAF (assuming a modestly large effect size), the missense variant in *DUOX2* in Family B was of interest given the established role of DUOX2 in CD. DUOX2 is a member of the large reduced nicotinamide adenine dinucleotide phosphate oxidase family of enzymes that are defective in chronic granulomatous disease.^43^ Knockdown of the DUOX2 homologue in invertebrates and mice results in an impaired tolerance to enteric bacteria.^54^ DUOX2 is overexpressed in intestinal biopsy specimens from CD patients associated with alterations in the intestinal microbiome.^55^ Two very rare functional mutations in *DUOX2* were recently identified in 2 patients with very early onset IBD in a candidate gene study.^56^ Of relevance given the overlap in cases with both *NOD2* and *DUOX2* variants in Family B, these 2 proteins have been shown to interact (mediated via the leucine-rich repeat domain of NOD2 in which CD-associated variants cluster) to protect cells from bacterial invasion.^57^ Genetic variation affecting the function of these proteins could alter CD risk through disrupting their interaction or by independently modulating their actions in host defense. Given the low frequency of the *DUOX2* and *NOD2* variants, replicating this association would require very large sample sizes. The observed *DUOX2* variant (P303R) impairs its function; however, even with functional data in the absence of statistical replication of the association of the variant to CD, we cannot infer causality.^58^

On the basis of existing functional data, a number of other prioritized candidate variants are worthy of note. NLRP2 is a NOD-like receptor and a component of the inflammasome; reducing endogenous levels of the protein has been shown to reduce lipopolysaccharide-induced secretion of IL1β in monocytes,^59^ which is of relevance given the defective proinflammatory cytokine secretion observed from CD macrophages.^51^, ^60^ ZC3H18 is involved in IκB kinase and nuclear factor-κB activation,^61^ a pathway of established importance in CD, and MEGF10 is a phagocytic receptor involved in apoptosis.^62^ However, as per the *DUOX2* variant, robust statistical evidence of disease association remains of overarching importance.^58^

We have assumed that each variant acts independently. However, it is possible that a selection of the prioritized candidate variants identified act in consort. For example, on a background of *NOD2* variants impairing immune cell activation,^63^ a DUOX2 variant altering mucosal immune homeostasis could cause CD. The problem with this model, as is the case for epistatic interactions of common variants underlying truly polygenic traits,^64^ is the difficulty in achieving statistical power to detect an association resulting from the requirement for the carriage of multiple rare variants. Furthermore, it is possible that novel risk variants preferentially affect those individuals with a low GRS^23^ and that this could be used for variant prioritization.

Familial aggregation does not necessarily imply an underlying genetic etiology. Familial clustering of IBD has been proposed to be environmental.^65^ However, in the families under study a primary environmental etiologic explanation is unlikely. If the disease was caused by an environmental factor, one would expect it to manifest equally frequently in all those living within the same household. However, across all nuclear families from Families A and B with cases, there was only 1 occurrence of a dually affected couple. In addition, the cases were distributed across multiple cousinships within different cities worldwide; it is difficult to envisage the propagation (or coincidental occurrence) of an environmental risk factor across these distances. Finally, the highly variable familial burden of disease observed in this population in the context of its environmental homogeneity argues in favor of a genetic etiology. It is possible, however, that an environmental factor (or factors) contribute to a reduced penetrance, for example, a genetically susceptible individual may develop the disease only after an environmental insult (eg, acute gastroenteritis^66^) that could occur stochastically.

This study has shown the complexity of the genetics of CD in 2 large AJ families with multiple cases of the disease. The role of common CD-associated variants has been highlighted and a monogenic etiology has been excluded. A number of candidate variants have been prioritized, most notably a novel frameshift mutation in the gene *CSF2RB* for which independent replication evidence has been obtained. The identification of this mutation and its independent discovery by Chuang et al^44^ consolidates the causal role of this mutation in CD and validates the approach of using exome sequencing in large families. Further candidate variants identified in this study may be implicated in CD; showing this will rely on further genetic and functional investigations. Regarding our understanding of CD pathogenesis, the identification of a mutation in CSF2RB, a protein common to multiple cytokine receptors, reinforces the importance of the innate immune system as a first defense against penetration of the microbiome through the intestinal mucosa.^52^
