# Supplementary material for: Genetic Complexity of Crohn’s Disease in Two Large Ashkenazi Jewish Families
Source: Gastroenterology. 2016 Oct;151(4):698–709. doi: 10.1053/j.gastro.2016.06.040 (PMC5643259; doi:10.1053/j.gastro.2016.06.040)
Supplement: Supplementary Data [file mmc4.pdf]

Genetic Complexity of Crohns Disease in 2 Large Ashkenazi Jewish Families

Supplementary Information

Adam P. Levine, Nikolas Pontikos, Elena R. Schiff, Luke Jostins, Doug Speed, NIDDK Inflammatory Bowel Disease Genetics Consortium, Laurence B. Lovat, Jeffrey C. Barrett, Helmut Grasberger, Vincent Plagnol and Anthony W. Segal

Correspondence: t.segal@ucl.ac.uk

Contents

1 Supplementary Methods 3
1.1 Ancestry assessment 3
1.2 Marker set for linkage analysis 3
1.3 Linkage analysis 3
1.4 Samples used for exome sequencing 3
1.5 Haplotype flow reconstruction 4
1.6 Within-family imputation 5
1.7 Candidate variant genotyping 5
1.8 DUOX2 functional experiments 6
1.8.1 Plasmids 6
1.8.2 Cell culture and transfection 6
1.8.3 Hydrogen peroxide production assay 6
1.8.4 Superoxide release assay 7
1.8.5 Flow cytometry 7

2 Supplementary Figures 9

3 Supplementary Table Legends 13

4 References 15

1 Supplementary Methods

1.1 Ancestry assessment

The Ashkenazi Jewish (AJ) ancestry of all individuals was confirmed by principal component analysis (PCA) using snpStats (v1.14.0 [1]) with a reference dataset of 471 unrelated individuals with four AJ grandparents [2] and non-Jewish populations (CEU (Utah residents with North and Western European ancestry) and TSI (Toscani in Italy)) from HapMap [3]. Related individuals and poorly genotyped samples and SNPs were removed. Common SNPs were extracted and pruned for LD (r^2 <0.2) in each dataset separately.

1.2 Marker set for linkage analysis

AJ specific RAFs for linkage were obtained using SNP data in 1,502 individuals of AJ ancestry, confirmed by PCA (as above) and extracted from a larger cohort on dbGAP [4] (phs000448.v1.p1) [5]. SNPs shared with those genotyped in the families were pruned for LD in the reference data at r^2 <0.2. The heterozygosity and RAF were computed using PLINK and the SNP with the highest heterozygosity sequentially within sliding windows of 0.1 and 0.5 cM were selected for the linkage map using a custom Python script

1.3 Linkage analysis

Linkage analysis was performed using Switflink. To account for the unknown disease penetrance, only affected individuals were consdiered. The Switflink MCMC was parallelised on four core-processors using default parameters and the average of ten replicates taken.

1.4 Samples used for exome sequencing

In addition to the affected individuals from the families, exome sequencing was undertaken on from a selection of unaffected family members and AJ controls. Specifically, in Family A, 23 unaffected family members were sequened comprising eight non-founder parents of affected individuals, six founders with no affected children and nine founders with one or more affected children. In Family B, 18 unaffected family members were sequenced comprising two non-founder parents of affected individuals, four founders

with one or more affected children and 12 unaffected siblings or cousins of affected individuals. In addition, 31 unrelated AJ controls were sequenced.

1.5 Haplotype flow reconstruction

The full pedigrees, a total of 322 individuals in Family A and 132 individuals in Family B were manually divided into non-overlapping subfamilies of ≤ 28 bits (twice the number of founders minus the number of non-founders) such that each subfamily was within the computational capabilities of Merlin [6]. For Family A there were 21 subfamilies and for Family B, seven. Within each subfamily, the most likely pattern of gene flow was estimated by Merlin using the 0.1 cM SNP map. For each non-founder within each subfamily, the founder source of each allele for each marker was thus determined. However, as the maternal/paternal classification of founder alleles is random (as the parents are unobserved) reassembling the split haplotype flow data is not straightforward. This is further complicated when founders are ungenotyped. To achieve haplotype founder source matching, hypotheses representing the different haplotype matching scenarios may be tested by comparing the sum of the observed probabilities of identical-by-descent inheritance for all individuals sharing each haplotype for each marker with that expected assuming they do indeed match. Pairwise identical-by-descent probabilities for each marker were estimated across all pairs of individuals in each full pedigree using a multiple splitting approach similar to that described by Thomson et al. [7]. Sub-pedigrees for all $x(x-1)/2$ pairs of individuals across the entire (pre-split) pedigree containing just the two individuals of interest, one genotyped sibling (if available, to assist with phasing) and their connecting ancestral relatives were generated; a total of 33,411 sub-pedigrees for Family A and 4,465 for Family B. Identical-by-descent probabilities using these sub-pedigrees were estimated using Merlin. In cases in which pairs of individuals appeared in multiple sub-pedigrees, the average identical-by-descent probabilities per marker across all observations of that pair of individuals was computed. Utilising these probabilities, haplotype matching was performed across the subfamilies progressively building up by adding one subfamily at a time. The maximum number of affected individuals sharing a founder haplotype within a particular family or subfamily was subsequently computed. These results were verified using Combinatorial Conflicting Homozygosity analysis [8].

1.6 Within-family imputation

For each variant, the imputation proceeded by first assigning the founder haplotypes for all wild type and homozygote individuals as reference and

alternate, respectively. Next, all heterozygote individuals were considered and if one of their founder haplotypes was reference or alternate, the other founder haplotype was assigned as the opposite, respectively. For each variant, this was repeated until no more founder haplotypes were updated. Consistency checks were performed at each step to verify that the two founder haplotypes and genotypes for each individual were compatible. If the allele frequency of the variant in ExAC and in the AJ control data (AJex) was <0.01, it was assumed that unobserved founder haplotypes would be wild type for the variant. Finally, the genotypes of all individuals for which both founder haplotypes were known were imputed. A similar approach has been described by Song et al. [9]. When an imputation conflict arose (in which the imputed genotype differed from that observed by direct genotyping for those individuals from whom it was available or when a haplotype was assigned as harbouring both the reference and alternate alleles), all imputed genotypes for that variant were discarded and only the genotypes in those individuals directly sequenced or genotyped were retained.

1.7 Candidate variant genotyping

The *DUOX2* and *CSF2RB* variants were genotyped in a selection of sequenced and imputed individuals for validation purposes. This was done by Sanger sequencing following PCR amplification of the flanking sequence. For the CSF2RB variant (chr22:37333972 GC/G, p.S709LX22), the forward primer was GTGGGAGGACAGGACCAAAA and the reverse was GGGAACTAGGGAGACAGACG yielding a product of 150 bp. For the DUOX2 variant (chr15:45402883 G/C, rs151261408, p.P303R), the forward primer was GCTGGAGAGATTTCCCTACTAAGC and the reverse primer was TCCTGTCTGAGTTGCTTCTCC yielding a product of 600 bp. In both cases, PCR was conducted using an annealing temperature of 60°C.

1.8 DUOX2 functional experiments

1.8.1 Plasmids

The c.908C>G (p.P303R) DUOX2 variant was introduced into an N-terminal hemagglutinin epitope (HA)-tagged DUOX2 expression vector [10] by site-directed mutagenesis (QuikChange; Stratagene, La Jolla, CA) (sense primer: 5'-CTGTGTATGAGTGGCTGCgCAGCTTCCTGCAGAAAACAC-3'). To generate expression vectors for FLAG-epitope tagged 303P and 303R DUOX2, the HA-tag was replaced by a FLAG (DYKDDDDK) encoding sequence using the splicing-by-overlap PCR technique with either 303P or 303R HA-DUOX2 plasmids as template. Internal primers in the first-round PCR reac-

tions were 5'-gactacaaggacgacgatgacaagGCACTCTCACTGCCCTGGGA-3' and 5'-cttgtcatcgtcgtccttgtagtcGTCCTGACTGCCCGATGGA-3' (FLAG encoding sequence in lower case). The products of the fusion PCRs were directionally cloned into the KpnI and PshAI sites of the HA-DUOX2 plasmid. The DUOXA2, DUOXA1 and DUOXA2-EGFP expression vectors were prepared as previously described [10]. All constructs were verified by bidirectional DNA sequencing.

1.8.2 Cell culture and transfection

HEK 293 were maintained in DMEM (Life Technologies, Carlsbad, CA, USA) supplemented with 10% heat inactivated fetal bovine serum. Adherent cells were transfected at 50-60% confluence using FuGENE 6 reagent (Promega, Madison, WI, USA). Plasmids encoding DUOX2 maturation factor (either DUOXA2 or DUOXA2-EGFP) were cotransfected at 13 ng/cm^2 of cell monolayer, whereas the amount of DUOX2 encoding plasmids was varied from 2.1-21 ng/cm^2. Under these conditions, DUOXA2 is available in significant excess and does not limit DUOX2/DUOXA2 heterodimerization [11,12]. In all experiments, the total amount of DNA transfected per square centimeter of cell monolayer was kept constant by adjusting with empty pcDNA3.1 vector.

1.8.3 Hydrogen peroxide production assay

Release of hydrogen peroxide was determined by reaction with cell-impermeable 10-acetyl-3,7-dihydroxyphenoxazine (Amplex Red reagent; Life Technologies) in the presence of excess peroxidase, producing fluorescent resorufin. Briefly, cell monolayers in 24-well plates were incubated in HBSS/10 mM Hepes (pH 7.4) supplemented with 50 µM Amplex Red reagent and 0.1 U/ml horseradish peroxidase for one hour at 37°C. For stimulation of DUOX2 NADPH-oxidase activity, ionomycin (1 µM) and 12-O-tetradecanoylphorbol-13-acetate (TPA; 400 nM) were included in the reaction buffer. Fluorescence (ex/em, 530/595 nm) of the medium was measured within the linear range of the hydrogen peroxide concentration response curve and corrected for Amplex Red oxidation in wells containing cells transfected with empty pcDNA3.1 vector only. As internal control for transfection efficiency, *Renilla* luciferase activity from cotransfected pRL-Tk plasmid (2 ng/well) was determined in the remaining cells.

1.8.4 Superoxide release assay

Extracellular superoxide release of cells resuspended in Krebs-Ringer-HEPES buffer (pH 7.4) was detected using Diogenes reagent (National Diagnostics, Atlanta, GA, USA). Chemiluminescence was recorded following the addition of TPA/ionomycin to 2×10^5 cells/200 µl reaction. Cells co-transfected with DUOX2 and DUOXA1 plasmids were used as positive control for O_2^- release [13,14]. Specificity of the assay for superoxide was ascertained by including superoxide dismutase (10 µg/ml) in parallel reactions.

1.8.5 Flow cytometry

To detect surface-expressed epitopes, cells were washed twice in PBS, and incubated for 30 min (15 min at room temperature, 15 min on ice) with either rat anti-HA (0.5 ng/ul of clone 3F10, Roche) or mouse anti-FLAG (1 ng/µl of clone M2; Sigma) diluted in HBSS/10 mM HEPES pH 7.4/1% BSA. Cells were washed twice in cold PBS, detached in 2 mM EDTA and fixed at 4°C in 0.75% formaldehyde. Bound antibodies were detected using Alexa Fluor 647-conjugated anti-rat or anti-mouse IgG, respectively. Cytometry data for 100,000 events per sample were acquired on a BD Accuri C6 Flow Cytometer (BD Biosciences) and appropriate FSC/SSC gates were employed to exclude cellular debris. Data were analyzed using FlowJo 8.8.7 software. Relative surface expression of DUOX2 was determined by calculating differences in total fluorescence intensity between the samples and an equal-sized population of control cells overexpressing DUOX2 but not its maturation factor. Without the latter, DUOX2 could not be detected in non-permeabilized cells (Figure 4D). For detection of intracellular DUOX2, detached cells were first fixed in 0.75% formaldehyde/PBS at 4°C, then washed and permeabilized with 0.2% saponin in PBS/0.1% BSA. Binding of antibodies was done as above, but in the presence 0.2% saponin. Total DUOX2 expression was determined by calculating differences in total fluorescence intensity between the samples and an equal-sized population of control cells transfected with empty pcDNA3.1 plasmid. To test whether expression of the 303R variant interferes specifically with the surface expression of wild type (303P) DUOX2, cells were transfected with equal amounts of HA-tagged and FLAG-tagged DUOX2 constructs of either the 303P and/or 303R variants and the surface expression of the HA-tagged variant determined under each condition. Surface expression of the FLAG-tagged DUOX2 constructs is depicted in Supplementary Figure 4.

2 Supplementary Figures

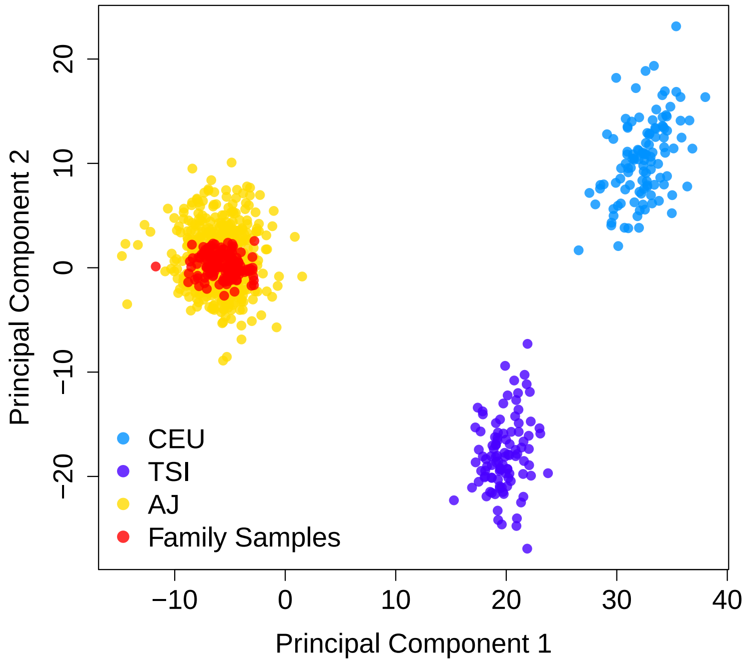

Figure S1: Principal component analysis showing the distinct separation of a reference panel of Ashkenazi Jewish (AJ) individuals from North and West European (CEU) and Tuscan (TSI) HapMap populations and the clustering of samples from the two families with the reference AJ population.

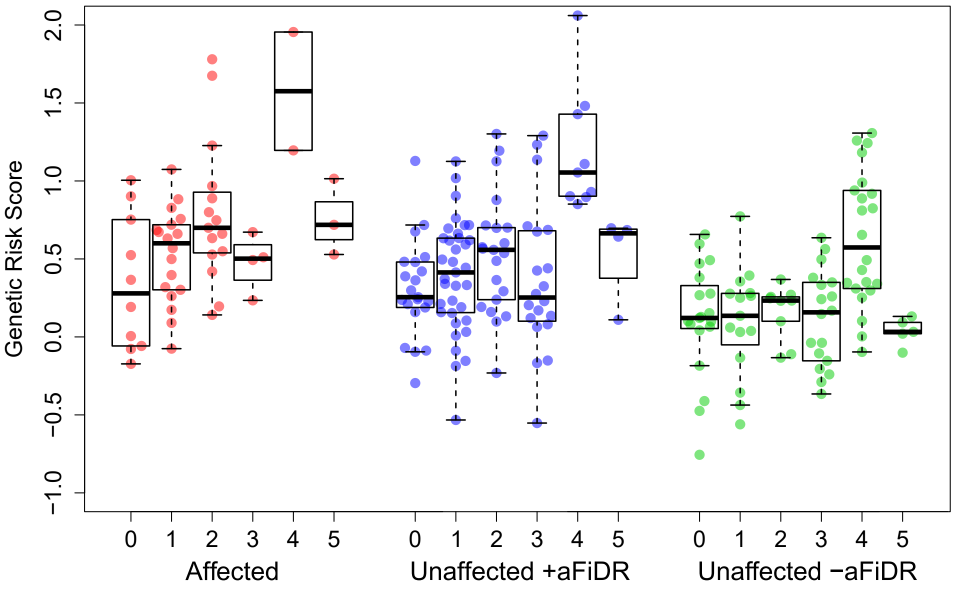

Figure S2: Genetic risk scores (GRS) in affected and unaffected individuals in Family A grouped by subfamily (labelled 0 through to 5). Unaffected individuals have been divided into those with (+aFiDR) and without (-aFiDR) at least one affected first degree relative.

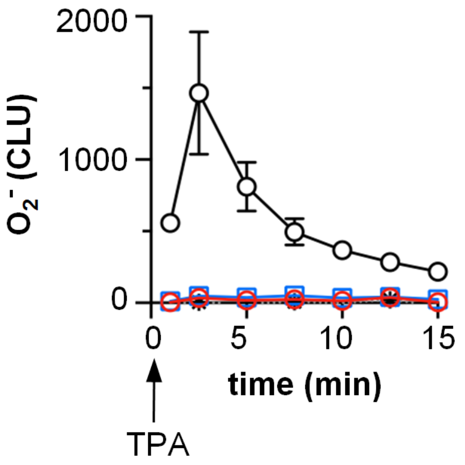

Figure S3: Chemiluminescence assay for superoxide (O_2^-) generation by the 303R-DUOX2 variant. Extracellular superoxide release was recorded following stimulation with TPA. As a positive control, DUOX2 was coexpressed with DUOXA1 to produce an unstable enzyme complex prone to O_2^- leakage [7,8]. 303R-DUOX2/DUOXA2 does not release detectable level of O_2^- consistent with normal dismutation of O_2^- to H_2O_2 within the enzyme complex. Data shown are form a single experiment and representative for the results of three independent experiments.

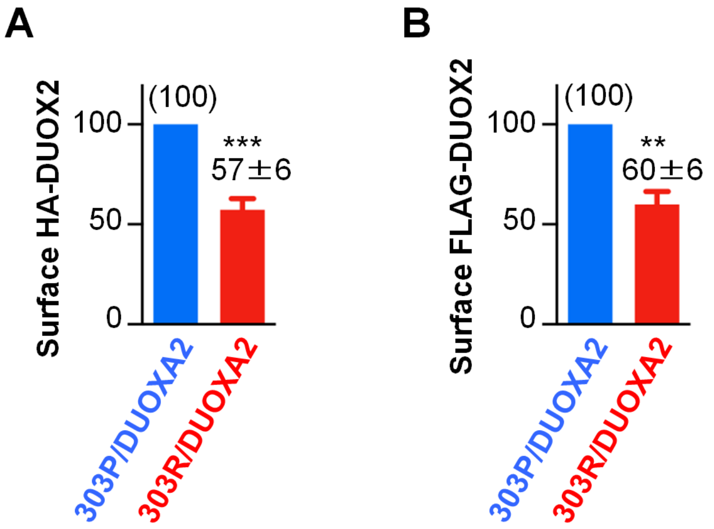

Figure S4: Cell surface expression of recombinant DUOX2 variants detected using HA (A) or FLAG (B) epitopes. Surface expression in transfected HEK293 cells was determined by flow cytometry of non-permeabilized cells. For each experiment, the values for the 303R-DUOX2 variant were normalized to those for wild type (303P) DUOX2 (set to 100%). Bars indicate mean(± SD) from n=4 independent transfection experiments. *** $p<0.001$, ** $p<0.01$ (ratio paired t-test).

3 Supplementary Table Legends

Supplementary Table 1
Association analysis (correcting for relatedness) results for 127 CD-associated risk variants in Family A and Family B. Column headers are as follows:

rsID: dbSNP identifier, **CHR**: chromosome, BP: genome position (Build 37), **RiskAllele**: Crohn's disease associated risk allele, **OR**: published odds ratio (see main paper for source), **Freq**: published frequency of the risk allele in controls (see main paper for source), **A_ControlFreq**: frequency of risk variant in unaffected individuals in Family A, **A_CaseFreq**: frequency of risk variant in affected individuals in Family A, **A_P**: LDAK p-value for Family A, **B _ControlFreq**: frequency of risk variant in unaffected individuals in Family B, **B_CaseFreq**: frequency of risk variant in affected individuals in Family B, **B_P**: LDAK p-value for Family B.

Supplementary Table 2
Exome variants prioritised in Family A (n=66). Column headers are defined in Table 1 of this document. The frequency of the variant in affected and unaffected individuals, LDAK p-value and direction of effect are shown for the family overall and for subfamilies A0, A1 and A2.

Supplementary Table 3
Exome variants prioritised in Family B (n=11). See legend for Supplementary Table 2.

**Field**	**Definition**
CHR	Chromosome
POS	Genome positions (Build 37)
REF	Reference allele
ALT	alternative allele
ID	rs ID, if available
Gene	Ensembl gene identifier
Feature	Ensembl transcript identifier(s)
Consequence	Variant consequence
cDNA_pos	cDNA position of alteration
Protein_pos	Protein position of alteration
Amino_acids	Amino acid change
SYMBOL	Gene symbol
CAROL	deleteriousness prediction
Condel	deleteriousness prediction
CADD	deleteriousness prediction
EXAC_Adj	ExAC composite allele frequency
ImmunoBase_CRO	Crohn's disease GWAS loci
UCLEX	UCLEX allele frequency.
BroadAJControls	AJex control Ashkenazi Jewish allele frequency
ONEGK_EUR	1000 Genomes, European allele frequency
freq cases	frequency in cases in the family (or subfamily)
freq controls	frequency in controls in the family (or subfamily)
effect	direction of effect in the family (or subfamily)
P	LDAK p-value in the family (or subfamily)
minP	Minimum p-value observed (A only)
family	Subfamily/family in which minP was observed (A only)
CONFLICT	Number of imputation conflicts observed
AJex_control_AF	AJex control allele frequency
AJex_case_AF	AJex case allele frequency
AJex_OR	AJex odds ratio
AJex_p	AJex p-value

Table 1: Definitions of columns in Supplementary Tables 2 and 3

4 References

1. Clayton D, Leung H-T. An R package for analysis of whole-genome association studies. Hum. Hered. 2007;64:4551.

2. Bray SM, Mulle JG, Dodd AF, et al. Signatures of founder effects, admixture, and selection in the Ashkenazi Jewish population. Proc. Natl. Acad. Sci. U. S. A. 2010;107:1622216227.

3. Altshuler DM, Gibbs RA, Peltonen L, et al. Integrating common and rare genetic variation in diverse human populations. Nature 2010;467:528.

4. Tryka KA, Hao L, Sturcke A, et al. NCBIs Database of Genotypes and Phenotypes: dbGaP. Nucleic Acids Res. 2014;42:D9759.

5. Guha S, Rosenfeld JA, Malhotra AK, et al. Implications for health and disease in the genetic signature of the Ashkenazi Jewish population. Genome Biol. 2012;13:R2.

6. Abecasis GR, Cherny SS, Cookson WO, et al. Merlin–rapid analysis of dense genetic maps using sparse gene flow trees. Nat. Genet. 2002;30:97101.

7. Thomson R, Quinn S, McKay J, et al. The advantages of dense marker sets for linkage analysis with very large families. Hum. Genet. 2007; 121:459468.

8. Levine AP, Connor TMF, Oygar DD, et al. Combinatorial Conflicting Homozygosity (CCH) analysis enables the rapid identification of shared genomic regions in the presence of multiple phenocopies. BMC Genomics. 2015;16:163.

9. Song S, Shields R, Li X, et al. Joint analysis of sequence data and single-nucleotide polymorphism data using pedigree information for imputation and recombination inference. BMC Proc. 2014;8:S20.

10. Grasberger H, Refetoff S. Identification of the maturation factor for dual oxidase. Evolution of an eukaryotic operon equivalent. The Journal of Biological Chemistry. 2006; 281:18269-72

11. Grasberger H, De Deken X, Miot F, Pohlenz J, Refetoff S. Missense mutations of dual oxidase 2 (duox2) implicated in congenital hypothyroidism have impaired trafficking in cells reconstituted with duox2 maturation factor. Molecular Endocrinology. 2007;21:1408-21

12. Rigutto S, Hoste C, Grasberger H, Milenkovic M, Communi D, Dumont JE, Corvilain B, Miot F, De Deken X. Activation of dual oxidases duox1 and duox2: Differential regulation mediated by camp-dependent

protein kinase and protein kinase c-dependent phosphorylation. The Journal of Biological Chemistry. 2009;284:6725-34

13. Zamproni I, Grasberger H, Cortinovis F, Vigone MC, Chiumello G, Mora S, Onigata K, Fugazzola L, Refetoff S, Persani L, Weber G. Biallelic inactivation of the dual oxidase maturation factor 2 (duoxa2) gene as a novel cause of congenital hypothyroidism. The Journal of Clinical Endocrinology and Metabolism. 2008;93:605-10

14. Morand S, Ueyama T, Tsujibe S, Saito N, Korzeniowska A, Leto TL. Duox maturation factors form cell surface complexes with duox affecting the specificity of reactive oxygen species generation. FASEB Journal.2009;23:1205-18
